# Evaluation of Serum and Urine Amino Acids in Dogs with Chronic Kidney Disease and Healthy Dogs Fed a Renal Diet

**DOI:** 10.3390/metabo11120844

**Published:** 2021-12-06

**Authors:** Marcio Antonio Brunetto, Doris Pereira Halfen, Larissa Wunsche Risolia, Vivian Pedrinelli, Douglas Segalla Caragelasco, Thiago Henrique Annibale Vendramini, Julio César de Carvalho Balieiro, Cristiana Fonseca Ferreira Pontieri, Juliana Toloi Jeremias, Bruna Ruberti, Marcia Mery Kogika

**Affiliations:** 1 Pet Nutrology Research Center, Department of Animal Nutrition and Production, School of Veterinary Medicine and Animal Science, University of São Paulo, Jardim Elite, Pirassununga 13635-900, Brazil; thiago.vendramini@usp.br (T.H.A.V.); balieiro@usp.br (J.C.d.C.B.); 2 Veterinary Nutrology Service, Veterinary Teaching Hospital, School of Veterinary Medicine and Animal Science, University of São Paulo, Cidade Universitária, São Paulo 05508-270, Brazil; dorisph2@yahoo.com.br (D.P.H.); larissa.risolia@usp.br (L.W.R.); vivian.pedrinelli@usp.br (V.P.); 3 Small Animal Internal Medicine Service, Veterinary Teaching Hospital, School of Veterinary Medicine and Animal Science, University of São Paulo, Cidade Universitária, São Paulo 05508-270, Brazil; mv.douglas@yahoo.com.br (D.S.C.); brunaruberti@usp.br (B.R.); mmkogika@usp.br (M.M.K.); 4 Nutrition Development Center, Grandfood Industry and Commerce LTDA (Premier Pet®), Dourado, São Paulo 13590-000, Brazil; cristiana@premierpet.com.br (C.F.F.P.); jjeremias@premierpet.com.br (J.T.J.)

**Keywords:** canine, nutrition, nephropathy, protein metabolism, renal failure

## Abstract

This observational study aimed to evaluate serum and urinary amino acid (AA) concentrations in healthy dogs and dogs with chronic kidney disease (CKD) fed a commercial therapeutic renal diet with reduced protein and phosphorus levels. Ten dogs with CKD stages 3 or 4 composed the study group and received the renal diet for 180 days (RG T180). A control group (CG T30) composed of seven healthy dogs was fed a renal diet for 30 days. When comparing serum AA between RG T180 and CG T30, histidine, isoleucine, leucine, lysine, phenylalanine, tryptophan, cysteine, citrulline, ornithine, taurine, branched-chain amino acids (BCAA), and total essential amino acids (EAA) were higher in RG T180. Meanwhile, arginine, asparagine, aspartate, glutamine, serine, and tyrosine were higher in CG T30. Serum phenylalanine, tryptophan, and hydroxyproline were higher in RG T0 (dogs with CKD before consuming a renal diet) when compared to RG T180. In addition, the serum ratios of arginine/citrulline, tyrosine/phenylalanine, and serine/glycine were higher in CG T30 than in RG T180. Concerning urinary AA concentrations in CKD dogs, isoleucine, phenylalanine, tryptophan, aspartate, cysteine, and BCAA were higher in RG T180. In urine, the total EAA/total non-essential AA ratio in RG T180 was higher than in CG T30 as well as tyrosine/phenylalanine ratio higher in CG T30. In conclusion, the combination of renal diet and conservative treatment over 6 months in dogs with CKD stages 3 or 4 affected the AAs metabolism when compared to healthy adult dogs.

## 1. Introduction

Chronic kidney disease (CKD) is characterized by renal dysfunction of one or both kidneys for an extended period, usually 3 months or longer. Its classical clinical and laboratory manifestations usually present when approximately 70% of nephrons are compromised. The renal damage is irreversible and often progressive, and it is estimated that 0.5 to 1.6% of dogs are affected by this condition [1,2]. More recent data suggest that about 7 to 10% of dogs between 8 and 10 years present with elevated SDMA or SDMA and creatinine concentrations, and the prevalence increases with age [3]. CKD causes a decrease in glomerular filtration rate as well as retention of various substances that are normally excreted by the kidneys, such as nitrogenous compounds (uremic toxins) and phosphorus.

The amino acids (AA) are essential to maintain functions of the organism, and the kidneys play a vital role in their homeostasis, metabolism, and serum concentration by reabsorption in the proximal tubules [4,5,6]. Kidney disorders can alter AA concentrations because the supply of AA to the kidneys is decreased by the reduced renal flow and glomerular filtration rate. Nevertheless, urinary amino acid clearances are often normal or increased [4].

Among the therapeutic approaches to CKD, it is recommended to provide renal diets for dogs stages 2 and up, and mainly for stage 3, classified based on serum creatinine concentrations according to the International Renal Interest Society (IRIS) [7]. The main objectives for providing a therapeutic renal diet include control of clinical signs, reduction of electrolyte and mineral disturbances, maintenance of body condition score (BCS) and muscle mass score (MMS), and supply of sufficient energy and essential nutrients [8]. Furthermore, previous studies have already demonstrated that the intake of a renal diet correlated to increased survival in dogs with CKD [8,9]. Therapeutic renal diets generally have reduced levels of protein and sodium and restricted levels of phosphorus compared to maintenance diets [10]. Clinical signs of CKD can be related to uremia, which leads to the assumption that protein-restricted diets could be beneficial to patients with CKD since protein is metabolized into urea and nitrogen compounds. In contrast, there is evidence that circulating and urinary AA metabolism changes in dogs with CKD [4,5], which brings to light if this protein restriction influences AA metabolism and clearance. These studies, however, did not standardize the diet consumed, and therefore, it was not possible to conclude if a therapeutic diet influences these changes.

Considering those points, it is essential to better understand how the disease affects the metabolism and excretion of AA, as well as to evaluate the impact of a renal diet on these animals. Thus, the present study aimed to evaluate the effects of a standardized therapeutic renal diet on serum and urinary AA concentrations in dogs with CKD stages 3 and 4 in comparison to healthy dogs.

## 2. Results

Serum urea, creatinine, total calcium, ionized calcium, phosphorus, parathormone (PTH), and fibroblast growth factor 23 (FGF-23) of groups RG T0, RG T180, and CG T30 are presented in Table 1. In general, serum urea, creatinine, and phosphorus were higher in the RG than CG, as expected, but serum creatinine increased between RG T0 and RG T180. There were no differences between the groups for total calcium and ionized calcium, and there were no differences in RG at T0 and T180 regarding PTH and FGF-23.

Serum total protein and albumin, BCS, MMS, and body weight (BW) of RG T0 and RG T180 are presented in Table 2. The only difference was in BW, which was lower in the RG T180.

Serum AA concentrations of RG at T0 and T180 were similar, except for phenylalanine, tryptophan, and hydroxyproline, which decreased over the time of diet consumption (Table 3). The same was observed for AA urinary analysis, in which only some AA have changed in RG throughout the disease progression and diet intake, such as taurine and total non-essential amino acids (NEAA) that decreased its concentrations at RG T180, and isoleucine increased at the same period. Differences in serum total essential amino acids (EAA) concentrations were observed in seven AA when compared to RG T180 and CG T30, and only methionine, threonine, and valine had similar concentrations in both groups. Urinary concentrations of EAA were similar between RG T180 and CG T30, except for isoleucine, phenylalanine, and tryptophan which were higher in RG T180.

Similar concentrations of each serum NEAA evaluation were observed between RG T0 and RG T180, and only hydroxyproline has decreased over time. Serum NEAA such as cysteine, citrulline, ornithine, taurine, branched-chain amino acids (BCAA), and total EAA were higher in RG T180 than in CG T30, the opposite of asparagine, aspartate, tyrosine, glutamine, and serine that were lower.

For urinary concentrations of NEAA, when comparing RG T180 and CG T30, only aspartate, cysteine, and total BCAA concentrations were higher in CKD dogs at RG T180.

Considering serum AA ratios, similar effects were observed between groups RG T180 and CG T30. The AA ratios between serine/glycine, tyrosine/phenylalanine, and arginine/citrulline were higher in CG T30 when compared with RG T180. The EAA/NEAA ratio was higher in CKD dogs (RG T0 and RG T180) when compared to CG T30.

## 3. Discussion

Dogs with CKD fed a therapeutic renal diet showed higher serum concentration of histidine, isoleucine, leucine, lysine, phenylalanine, tryptophan, cysteine, citrulline, hydroxyproline, ornithine, taurine, BCAA, and EAA than healthy control dogs fed the same diet. Moreover, lower concentrations of arginine, asparagine, aspartate, glutamine, serine, and tyrosine were observed in animals with CKD when compared to healthy animals.

The progression of CKD in humans increases the activity of the tryptophan’s catabolism enzyme due to the presence of chronic inflammation, resulting in the reduction of this AA in the bloodstream [8], which could be related to the restriction of protein intake. However, Hansen et al. [4] did not observe its reduction in dogs with CKD fed a diet with 16.0% of protein on a dry matter basis (similar to inclusion of 0.04 g/100 kcal). In the present study, serum concentrations of tryptophan decreased after 180 days of renal diet ingestion in dogs with CKD, which corroborates with the findings of Schefold et al. [11]. The urinary concentration of tryptophan, however, did not differ between RG T0 and RG T180, suggesting the diet rather than the metabolism could be responsible for the regulation [4,11]. The results also included a higher concentration of both serum and urinary tryptophan concentrations in RG when compared to CG. The decline in renal function may be the cause, but there may be some other alterations that could have influenced the concentration. Approximately 95% of the tryptophan is metabolized through the kynurenine pathway, regulated by the enzymes tryptophan 2,3-dioxygenase (TDO), present mainly in the liver, and indoleamine-(2,3)-dioxygenase (IDO), present in extrahepatic tissues [12]. However, there is little information regarding these two enzymes in dogs to conclude if they could have played a role in the increased tryptophan concentrations in RG.

This study demonstrated that serum taurine concentration was higher in RG T180 than the CG T30, and urinary taurine excretion was higher in RG T0 when compared to RG T180. This information corroborates with a study performed by Young [13], who observed that a reduced dietary intake of taurine may be a factor which contributes to taurine depletion in human treated with a low protein diet. The kidney plays an important role in regulating the storage of taurine [14]. The osmolarity regulating capacity is one of the most important biological properties of taurine, besides renal blood flow, glomerular filtration rate, osmoregulation, ion reabsorption and secretion, and composition of urine [4]. Therefore, the results of our study may indicate that dogs with CKD may have partially lost the renal ability to regulate taurine.

Another amino acid that had a difference between groups was methionine, which is an EAA that may be metabolized to cysteine by transmethylation (TM). This transformation has an intermediate AA, homocysteine, which is transsulfurized (TS) and generates cysteine. Besides TS, another alternative to homocysteine is remethylation (RM), which leads to methionine production. A higher serum concentration of cysteine was observed in RG, which may be related to the reduction of homocysteine clearance without the concomitant alteration of the TS reaction [15]. Regarding urine AA concentrations, cysteine concentration was higher in RG T180 when compared to CG, probably caused by higher serum concentration concomitant to glomerular filtration reduction. Methionine urine concentration was not altered between RG T0 and RG T180.

Serum hydroxyproline decreased in RG T180 when compared to RG T0. This amino acid is non-proteinogenic and is specific to collagen in mammals, and its serum concentration may reflect collagen metabolism [16]. Hydroxyproline is one of the NEAA which may be increased in the plasma of humans with CKD [17,18]. There is no information to date on hydroxyproline metabolism in dogs or cats with CKD; however, in humans, Laidlaw et al. [19] observed serum increase of plasma hydroxyproline in CKD patients in advanced stages, which differs from the results found in the present study. There was no difference between urinary hydroxyproline levels in both CKD and control groups, which suggests that maybe there may not be a correlation with the urine concentrations of hydroxyproline and CKD in dogs. However, serum concentrations of hydroxyproline in CKD dogs decreased after 180 days of diet intake, becoming similar to concentrations of the control group, suggesting that either the diet helped to control the serum metabolism of that AA or that there is another mechanism involving collagen that has not yet been clarified in dogs with CKD.

Another NEAA is glutamate, which is associated with the mechanism of acid–base balance. The kidney plays a fundamental role in the regulation of acid–base homeostasis through the excretion of urinary ammonium (NH_4_^+^) and the regeneration of bicarbonate. During metabolic acidosis, glutamine is converted to glutamate into the mitochondria of the proximal tubule cells. Glutamate is then metabolized by transamination and generates alpha-ketoglutarate. This substrate can be transformed by phosphoenolpyruvate carboxykinase (PEPCK) into glucose and two bicarbonate molecules [20]. These two mechanisms help to restore the systemic acid–base imbalance. In the present study, serum glutamine in RG T180 was numerically lower than in CG T30 dogs, but it was not statistically different, which leads to the conclusion that the diet might not have impacted the concentration of this amino acid.

When comparing RG T180 with CG T30, a higher serum concentration was observed of seven out of the 10 EAA. Considering that these AA are only found in the organism due to ingestion, which was standardized between both groups, those increases probably occurred due to clearance reduction. The concentrations of isoleucine, phenylalanine, and tryptophan were increased in the serum and urine of RG T180 animals while no changes in urine concentration were observed in the other EAA. It can be hypothesized that in patients with CKD, there is less metabolization and transformation to other amino acids or less requirement of these EAA. These results for EAA are different from those found by Parker et al. [5] when comparing healthy dogs to renal proteinuric dogs (RPD). The authors found lower concentrations of histidine, leucine, threonine, and total EAA. In addition, the authors did not observe increased concentration in any other EAA. These differences may have occurred due to the amount of food consumed by dogs. In our study, dogs ate 100% of the calculated amount to ensure the consumption of the daily energy requirement and EAA. It is possible that in the report from Parker et al. [5], the consumption of the total daily requirement of EAA was not enough as the amount of food ingestion was not measured. Furthermore, the composition of the food given was not controlled, and therefore, the amounts of AA were not standardized or quantified.

AA serum investigation detected a higher concentration of leucine, isoleucine, and total BCAA in RG T180, in comparison to CG T30. Ceballos et al. [21] reported that in advanced stages of CKD, a moderate decrease in circulating BCAA may occur due to malnutrition from the intake of an inadequate diet. The metabolism of BCAA is affected by caloric intake, and when it is insufficient, AA catabolism occurs preferentially from BCAA in the muscle [22]. In our study, during the observational period from RG T0 to RG T180, no alteration was reported in MMS. This may indicate muscle preservation and appropriate protein intake. In addition, plasma levels of BCAA (especially valine and leucine) have been correlated with the CKD patient’s nutritional status, indicating the nutritional adequacy of CKD dogs fed the renal diet. At last, normalization of BCAAs may reduce anorexia and improve energy and protein consumption [23] and can add more benefits to CKD dogs.

In addition to an, ratios were calculated to assess the ability of the kidneys to interconvert AA [24]. Between the initial time-point and after 180 days, there was no change in the arginine/citrulline ratio, but the serum serine/glycine ratio was higher in CG, and serum and urine tyrosine/phenylalanine ratios decreased in animals with CKD when compared to healthy animals. Those findings suggest a poor ability of the kidneys of dogs with CKD to interconvert these amino acids.

It was observed that patients with CKD had low plasma tyrosine concentration while the levels of phenylalanine did not change, which was responsible for the reduction of the tyrosine/phenylalanine ratio [25,26]. Tyrosine production in the body occurs by the hydroxylation of phenylalanine through the action of the phenylalanine hydroxylase enzyme (PHE). In a study with rats conducted by Wang et al. [27], it was observed that hepatic PHE activity was reduced when protein intake was limited causing a decrease in tyrosine plasmatic concentration. The reduction in PHE activity can be attributed to uremia or malnutrition [10]. These changes corroborate the findings of this study, as there was a higher concentration of phenylalanine in the RG T180 group with greater urinary excretion of this AA as well. Furthermore, it was noticed that there was a decrease in phenylalanine level in the RG after the ingestion of renal food, indicating that the renal diet may have influenced the phenylalanine serum reduction. Regarding the ratio between tyrosine and phenylalanine, lower serum concentration was observed in RG T180 when compared to CG T30, which was expected by the lack of adequate phenylalanine hydroxylation.

Regarding citrulline and arginine, the kidneys are the main site of their conversion, which may be altered in CKD [28,29]. Citrulline is produced in the intestine and is taken up by the kidneys and is a precursor of arginine production. The uptake of citrulline and the release of arginine are impaired in individuals with CKD [30]. Normally, plasma citrulline concentration is increased by deficient uptake, while arginine concentrations are usually maintained at normal levels because the diet can also be an arginine source [13]. Increased citrulline concentration in dogs with CKD was also observed by Hansen et al. [4] in a study evaluating dogs fed a protein-restricted diet. It was observed in our study that RG T180 showed lower serum concentrations of arginine than CG T30, indicating a possible inadequacy of the diet concerning this EAA, associated with a low conversion rate. Chen et al. [31] reported lower activity of the enzymes argininosuccinate synthetase and argininosuccinate lyase in rats with CKD, which are enzymes responsible for conversion of citrulline to arginine in the renal cortex which can also explain the results. There were no urinary changes in arginine, and citrulline was not detected, probably due to the deficit in the uptake capacity.

The serum EAA/NEAA ratio was increased in dogs with CKD. In urine, EAA/NEAA ratio was higher at RG T180 than at RG T0 and higher in the CG when compared to RG T180. Usually, patients with CKD show high plasma concentrations of NEAA and low concentrations of EAA [32,33]; however, the mechanisms of these abnormalities have not yet been fully elucidated [34]. In humans with CKD, there are lower concentrations of EAAs than in healthy patients as a consequence of low food ingestion [35,36]. This profile was not seen in our study, where dogs have eaten all the proposed amount of food.

This study had some limitations mainly regarding the animals who composed the groups and their ownership. The diet consumed by the dogs before the experiment was not controlled because client-owned dogs were involved. In addition, the study population was not completely homogenous (regarding age, weight, breed, and BCS), but as this was a prospective clinical study, it was accepted to ensure the sample size for the period of the study. Another limitation of this study is that the groups did not consume the diets for the same period (30 days vs. 180 days). As the diet provided in this study was a therapeutic renal diet, which has lower protein and especially phosphorus levels, the authors believed it should not be provided for healthy animals for more than 30 days to avoid nutritional deficiencies [37,38]. During the present study, some animals had to be treated with phosphate binders due to hyperphosphatemia. As there is no evidence to this date that phosphate binders can influence the serum or urine amino acid profile, the authors did include these animals in the study. Furthermore, as this was a clinical study with client-owned animals, hyperphosphatemia treatment was not postponed to ensure the long-term health of the animals included.

It is important to state that this was a pioneer observational study, as there are no studies that have evaluated the amino acid profile of dogs with CKD. This is also one of the few studies that standardized the diet, which is a parameter that could potentially influence the serum and urine amino acid profile aside from CKD. Further AA analysis that could be evaluated during disease progression has not been performed due to the assay’s high cost. Finally, increases in serum urea, creatinine, and phosphorus were observed among the RG, indicating disease progression which may have influenced AA excretion. Serum and urine AA levels may have been affected by the stage classification that the dogs with CKD were involved (some animals have advanced one stage on the disease classification, which means a decrease in renal function by creatinine evaluation). According to Kopple et al. [17], loss of functioning renal parenchyma causes renal impairment of metabolic functions, and then, many changes including those involving metabolism of AA could happen.

## 4. Materials and Methods

### 4.1. Animals and Study Site

This prospective study was conducted at the Veterinary Teaching Hospital of the School of Veterinary Medicine and Animal Science of the University of São Paulo (FMVZ/USP), São Paulo-SP, Brazil, and approved by the Ethics Committee of the School of Veterinary Medicine and Animal Science of the University of São Paulo (FMVZ/USP) on 4 September 2013, protocol number 3138/2013. The animals with CKD used in this study were client-owned dogs selected from the Service of Small Animal Internal Medicine of the Veterinary Teaching Hospital of FMVZ/USP between September 2013 and December 2015. The inclusion criteria for the renal group (RG) were adult dogs with persistent azotemia > 2.1 mg/dL for at least three months with a stable clinical condition and that did not consume a therapeutic renal diet. Exclusion criteria included animals with hiporexia or anorexia, emesis, diarrhea, or related clinical signs of gastric alterations and animals that had consumed or were consuming therapeutic renal diets. The healthy animals for the control group (CG) were selected from the kennel from Grandfood (Dourado, Brazil). The inclusion criteria for CG were animals deemed healthy by physical examination, complete blood count, and serum biochemistry profile, with no comorbidities and clinical signs of any sort. Two groups were set up according to the health status of dogs. The first one was the renal group (RG) and consisted of 10 dogs (5 males and 5 females) of various breeds (Golden Retriever, Labrador Retriever, Bulldog, German Shepherd, American Pit Bull Terrier, Cocker Spaniel, Beagle, Miniature Schnauzer, and 2 mixed-breed dogs), with a mean age of 8.89 ± 4.46 years and mean body weight of 16.33 ± 14.64 kg. These animals were diagnosed with CKD based on persistent azotemia and were classified as stages 3 or 4 according to IRIS [7]. In the urinalysis, these dogs did not show renal proteinuria. The animals from RG were kept in their households and taken care of by their owners, who complied with the study and signed an informed consent form, which stated that owners should not provide any other food other than the therapeutic diet provided. The animals from the CG were kept and cared for at the Grandfood kennel. During the experimental period, five dogs from RG were treated with phosphorus binder (aluminum hydroxide) as they had developed hyperphosphatemia. Moreover, RG animals showed stable serum concentrations of urea, and no clinical signs of decompensation such as anorexia or impairment of appetite, nausea/vomiting, or associated conditions. The second group was the control group (CG) and consisted of seven healthy adult mixed-breed dogs, with a mean age of 5.89 ± 2.57 years and a mean body weight of 8.74 ± 1.86 kg.

### 4.2. Diet

During the experimental period, the animals received a dry commercial diet for dogs with CKD (Table 4). The animals from the RG received the diet according to their estimated intake between the scheduled reassessments.

The amount of food supplied to each animal was determined according to the NRC energy requirement equation of 95 kcal × (kg of body weight)^0.75^/day [37] and animals were gradually adapted to the new diet for approximately one week. Periodic evaluations were performed for adjustments in the amount of food to ensure animals would maintain body weight throughout the experimental period.

**Figure 1:**
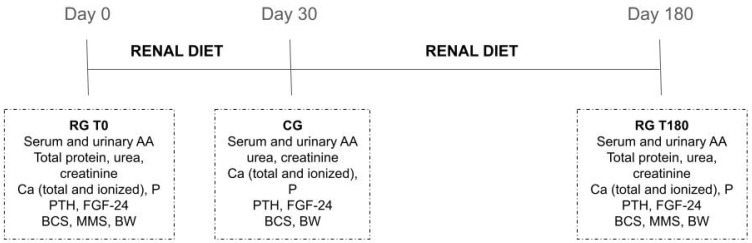
Visual scheme of the experimental schedule.

The RG received the diet for 180 days, and samples of blood and urine were collected at the beginning of the experimental period (RG T0) and 180 days after it (RG T180). The healthy dogs from CG received the renal diet for 30 days, and at the end of this period (CG T30), blood and urine samples were collected. Serum total protein and albumin, BCS, MMS, and BW were measured at RG T0 and RG T180. BCS and MMS were measured according to Laflamme [39] e Michel [40] and were always assessed by the same researcher (DPH). Serum urea, creatinine, calcium (ionized and total), phosphorus, parathormone, and FGF-23 were measured in RG T0, RG T180, and CG T30. A scheme of the schedule is depicted in Figure 1.

### 4.3. Sample Collection and AA Determination

Urine samples were collected by cystocentesis, guided by ultrasonography, or urethral catheterization. Samples were kept refrigerated and stored within 12 h after collection for AA measurements. Urinary density was established by refractometry, and physicochemical characteristics of urine were measured with reagent tapes (Combur-Test^®^, Roche, Basel, Switzerland). For blood collection, animals underwent a 12 h fasting period, and venous blood samples were collected and placed in a coagulation activator tube (BD Vacutainer^®^ SST ™ Advance, BD, Franklin Lakes, NJ, USA). The samples were centrifuged, and serum was obtained afterward. Amounts of 0.5 mL of serum and 1.0 mL of urine were stored at −80 °C until analysis. Samples were analyzed by high-performance liquid chromatography (HPLC) (Agilent 1200 Series, Santa Clara, CA, USA) on LUNA C18 100Å 5 u 250 × 4.6 mm 00G-4252-EQ column for determination of the total AA. Free AAs were analyzed on Luna 3u C18 (2) 100A 250 × 4.6 mm HPLC column 00G-4251-E0, based on the technique of White et al., 1986 [41]. The reading was performed at a wavelength of 254 nm. Diet AA profile was also determined by HPLC, and previous acid hydrolysis by the use of hydrochloric acid 6N has been performed for 24 h. All analyzes were performed in duplicate.

### 4.4. Statistical Analysis

Statistical analyzes were performed using the Statistical Analysis System (Version 8.2, SAS Institute Inc., Cary, NC, USA). Data were previously tested for normality by the Shapiro–Wilk test and homogeneity of the variances by the F test. Parametric data were analyzed by paired *t*-test (RG T0 vs. RG T180) and unpaired *t*-test (RG T0 vs. CG T30; RG T180 vs. CG T30). Non-parametric data were analyzed by the Wilcoxon test (corresponding to the paired *t*-test) or by the Mann–Whitney test (corresponding to the unpaired *t*-test). Values of *p* ≤ 0.05 were considered significant.

## 5. Conclusions

This study indicates a difference in AA metabolism between healthy dogs and dogs with CKD stages 3 and 4 when fed a renal diet for 6 months. An adaptation in the AA profile intake of dogs with CKD should be considered to ensure that these metabolic differences take a minimum effect on nutritional status. The authors suggest that to thoroughly investigate the AA metabolism of CKD dogs, further studies must be conducted.

## Figures and Tables

**Table 1:** Serum concentrations of urea, creatinine, total calcium, ionized calcium, phosphorus, parathormone, and fibroblast growth factor 23 (FGF-23) of dogs with chronic kidney disease before (RG T0) and after 180 days (RG T180) of therapeutic renal diet intake and healthy dogs (CG T30) after 30 days of therapeutic renal diet intake.

Variables	Treatments			RG T0 vs. RG T180		CG T30 vs. RG T180	
	CG T30	RG T0	RG T180	*p*	SEM ^2^	*p*	SEM ^2^
Urea (mg/dL)	39.29	206.07	235.34	0.1871	13.75	0.0002 ^1^	-
Creatinine (mg/dL)	1.12	3.11	4.30	0.0022	0.34	<0.0001	0.45
Total calcium (mg/dL)	10.73	11.33	11.94	0.3129	0.34	0.0847	0.34
Ionized calcium (mmol/L)	1.41	1.41	1.38	0.2327	0.2	0.3442	0.02
Phosphorus (mg/dL)	3.34	5.15	5.36	0.6309	0.28	<0.0001	0.30
Parathormone (pg/mL)	-	145.81	336.48	0.2077	70.62	-	-
FGF-23 (pg/mL)	-	5645.67	5788.56	0.8580	1157.67	-	-

^1^ Non-parametric test (Mann–Whitney); ^2^ SEM: standard error mean.

**Table 2:** Serum concentrations of total protein, albumin, body condition score (BCS), muscle mass score (MMS), and body weight of dogs with chronic kidney disease before (RG T0) and after 180 days (RG T180) of therapeutic renal diet intake.

Variables	Treatments		RG T0 vs. RG T180	
	RG T0	RG T180	*p*	SEM ^2^
Total protein (g/dL)	6.12	6.17	0.7772	0.12
Albumin (g/dL)	3.23	3.18	1.0000 ^1^	0.06
BCS	5.70	5.30	0.3750	0.20
MMS	2.20	2.40	0.6250 ^1^	0.10
Body weight (kg)	16.34	14.78	0.0453	3.05

^1^ Non–parametric test (Mann–Whitney); ^2^ SEM: standard error mean.

**Table 3:** Mean concentrations ± SD of serum and urinary amino acids in healthy dogs and dogs with chronic kidney disease stages 3 and 4 fed with a renal diet.

Variables	Serum					Urine				
	CG T30 ^1^	RG T0 ^2^	RG T180 ^3^	*p* RGT0 vs. RG T180	*p* RG T180 vs. CG T30	CG T30 ^1^	RG T0 ^2^	RG T180 ^3^	*p* RG T0 vs. RG T180	*p* RG T180 vs. CG T30
**Essential Amino Acids (µmol/L)**										
Arginine	367.78 ± 40.67	165.98 ± 24.06	153.36 ± 24.06	0.435	<0.0001	36.73 ± 6.51	31.75 ± 24.52	27.12 ± 12.70	0.568	0.284
Histidine	53.68 ± 6.25	65.02 ± 10.38	74.77 ± 15.64	0.079	0.001	8.20 ± 3.09	49.20 ± 52.41	56.05 ± 90.16	0.842	0.165
Isoleucine	15.76 ± 1.14	44.11 ± 12.97	45.83 ± 14.56	0.750	<0.0001	1.34 ± 0.59	23.17 ± 10.16	33.84 ± 13.84	0.049	<0.0001
Leucine	16.62 ± 2.79	87.65 ± 28.54	86.22 ± 25.39	0.893	<0.0001	1.27 ± 0.24	16.82 ± 11.62	21.08 ± 33.26	0.659	0.069
Lysine	33.34 ± 3.44	107.27 ± 27.14	110.51 ± 32.39	0.782	<0.0001	9.68 ± 1.28	32.84 ± 44.86	28.38 ± 19.56	0.751	0.233
Methionine	51.31 ± 11.45	44.79 ± 10.38	41.57 ± 9.92	0.500	0.071	6.73 ± 3.97	22.04 ± 19.58	23.20 ± 20.71	0.899	0.879
Phenylalanine	1.82 ± 0.90	76.64 ± 10.06	66.23 ± 8.37	0.007	<0.0001	1.74 ± 1.37	18.49 ± 13.25	27.89 ± 21.08	0.202	0.0028
Threonine	117.68 ± 46.39	150.57 ± 38.02	164.85 ± 82.04	0.599	0.123	13.21 ± 9.45	92.13 ± 146.79	35.99 ± 17.98	0.223	0.646
Tryptophan	10.27 ± 41.64	199.59 ± 30.00	150.66 ± 2.11	0.001	<0.0001	3.85 ± 2.51	78.25 ± 24.06	82.96 ± 23.48	0.614	<0.0001
Valine	133.33 ± 12.44	113.67 ± 32.56	122.68 ± 29.41	0.472	0.440	3.00 ± 1.17	14.77 ± 23.26	18.55 ± 35.97	0.759	0.245
**Non-Essential Amino Acids (µmol/L)**										
Alanine	322.79 ± 92.36	351.64 ± 123.89	361.34 ± 178.00	0.878	0.583	23.36 ± 5.19	157.17 ± 201.62	115.97 ± 75.83	0.527	0.189
Asparagine	100.97 ± 22.45	70.45 ± 22.45	72.30 ± 18.27	0.832	0.006	23.94 ± 4.97	53.19 ± 43.73	56.92 ± 45.18	0.837	0.107
Aspartate	15.05 ± 14.89	7.31 ± 3.76	6.25 ± 1.15	0.763	0.031	4.68 ± 0.64	19.45 ± 14.43	18.13 ± 12.08	0.800	0.026
Cysteine	1.28 ± 0.13	7.65 ± 3.46	7.90 ± 5.11	0.884	0.001	1.36 ± 0.75	15.44 ± 9.30	11.62 ± 6.28	0.278	0.011
Citrulline	10.64 ± 1.84	95.47 ± 21.55	117.23 ± 44.62	0.130	<0.0001	40.03 ± 9.92	Not detected	Not detected	-	-
Glycine	225.57 ± 60.48	253.75 ± 98.37	247.49 ± 114.45	0.886	0.651	22.04 ± 9.35	157.05 ± 181.38	96.37 ± 93.60	0.288	0.239
Glutamate	32.61 ± 9.27	27.59 ± 8.10	25.22 ± 6.24	0.502	0.066	6.76 ± 1.75	17.67 ± 12.26	14.82 ± 19.84	0.721	0.348
Glutamine	727.71 ± 111.12	506.40 ± 87.97	547.11 ± 161.42	0.475	0.007	33.83 ± 23.18	167.49 ± 261.62	116.65 ± 123.53	0.528	0.353
Hydroxyproline	11.27 ± 3.54	32.75 ± 22.51	16.35 ± 6.85	0.018	0.484	15.73 ± 5.49	53.09 ± 71.81	37.30 ± 14.50	0.460	0.359
Ornithine	6.39 ± 1.13	17.72 ± 5.95	15.15 ± 4.20	0.213	0.001	3.15 ± 0.55	9.24 ± 8.00	8.46 ± 3.49	0.780	0.081
Proline	127.03 ± 15.41	164.83 ± 73.24	133.27 ± 37.40	0.178	0.806	5.10 ± 1.92	91.89 ± 81.76	39.04 ± 30.97	0.095	0.235
Serine	124.98 ± 66.79	80.11 ± 30.26	74.92 ± 16.55	0.771	0.016	43.01 ± 12.44	94.36 ± 69.32	88.85 ± 59.98	0.829	0.112
Taurine	49.38 ± 186.50	333.30 ± 116.91	236.06 ± 17.64	0.120	0.009	1764.26 ± 381.01	2404.53 ± 1605.12	620.51 ± 919.43	0.003	0.057
Tyrosine	39.02 ± 9.85	33.11 ± 10.62	28.41 ± 6.08	0.252	0.024	2.40 ± 1.69	12.89 ± 14.40	8.28 ± 7.37	0.349	0.263
BCAA ^4^	165.71 ± 11.09	254.44 ±72.86	254.74 ± 67.68	0.736	0.006	5.43 ± 1.95	46.66 ± 3.57	65.30 ± 72.82	0.465	0.023
Total EAA ^5^	801.59 ± 77.76	1055.29 ± 170.79	1016.69 ± 163.89	0.570	0.007	85.60 ± 21.00	347.09 ± 308.55	280.09 ± 171.54	0.495	0.080
Total NEAA ^6^	1755.67 ± 227.14	1948.98 ± 520.03	1860.58 ± 457.51	0.656	0.632	1987.32 ± 419.52	2935.81 ± 1756.05	1097.30 ± 934.51	0.002	0.157
**Amino Acids Ratio**										
EAA ^5^/NEAA ^6^	0.46 ± 0.04	0.56 ± 0.09	0.57 ± 0.13	0.853	0.032	0.04 ± 0.01	0.17 ± 0.14	0.42 ± 0.32	0.012	0.001
Valine/Glycine	0.63 ± 0.22	0.50 ± 0.18	0.59 ± 0.31	0.391	0.735	0.15 ± 0.05	0.09 ± 0.08	0.14 ± 0.12	0.220	0.980
Arginine/Citrulline	29.05 ± 3.66	1.80 ± 0.57	1.48 ± 0.59	0.694	<0.0001	0.95 ± 0.25	-	-	-	-
Tyrosine/Phenylalanine	36.94 ± 47.07	0.43 ± 0.11	0.43 ± 0.08	1.000	0.004	2.93 ± 1.92	0.68 ± 0.33	1.03 ± 1.66	0.011	0.062
Serine/Glycine	0.65 ± 0.59	0.32 ± 0.07	0.33 ± 0.07	0.966	0.041	2.05 ± 0.37	1.12 ± 0.97	1.47 ± 1.55	0.109	0.310

^1^ CG T30: control group after 30 days of renal diet consumption; ^2^ RG T0: renal group before renal diet consumption; ^3^ RG T180: renal group after 180 days of renal diet consumption; ^4^ BCAA: branched-chain amino acids; ^5^ EAA: essential amino acids; ^6^ NEAA: non-essential amino acids.

**Table 4:** Chemical composition in percentage of dry matter and amino acid profile of the commercial renal diet used ^1^ and National Research Council [37] and European Pet Food Industry Federation [38] recommendations.

Nutrient	Diet	NRC	FEDIAF
Dry matter (%)	90.00	-	-
Crude protein (%)	14.50	10.00	21.00
Fat (%)	18.00	5.50	5.50
Ash (%)	5.50	-	-
Crude fiber (%)	3.50	-	-
Calcium (mg/kg)	0.50	0.40	0.58
Phosphorus (mg/kg)	0.30	0.30	0.46
**Essential Amino Acids (g/100 g)**			
Arginine	0.93	0.35	0.60
Phenylalanine	0.63	0.45	0.63
Histidine	0.34	0.19	0.27
Isoleucine	0.60	0.38	0.53
Leucine	1.08	0.68	0.95
Lysine	0.90	0.35	0.46
Methionine	0.65	0.33	0.46
Threonine	0.49	0.43	0.60
Tryptophan	0.17	0.14	0.20
Valine	0.75	0.49	0.68
**Non-Essential Amino Acids (g/100 g)**			
Alanine	1.07	-	-
Aspartate	1.25	-	-
Cysteine	0.07	-	-
Glycine	1.02	-	-
Glutamate	2.13	-	-
Proline	0.52	-	-
Serine	0.76	-	-
Taurine	-	-	-
Metabolizable energy (Kcal/g)	4.072	-	-

^1^ Premier Nutrição Clínica Renal Cães. Ingredients: Poultry by-product meal, isolated soy protein, powdered egg, rice bran, whole corn, barley, beet pulp, poultry fat, stabilized animal fat, fish oil, chicken hydrolyzate, BHA antioxidant, potassium citrate, potassium chloride, dry brewer’s yeast, transchelated mineral premix (copper, iron, iodine, manganese, selenium, and zinc), vitamin premix (folic acid, pantothenic acid, biotin, choline, niacin, pyridoxine, riboflavin, thiamine, vitamin A, vitamin B12, vitamin C, vitamin D, and vitamin E).

## Data Availability

The data presented in this study are available on request from the corresponding author.
